# Contemporary Characteristics and Outcomes of Pediatric Oncology Patients Participating in Early Phase Clinical Trials

**DOI:** 10.1002/cam4.71222

**Published:** 2025-09-08

**Authors:** Avina Rami, Kira Bona, Suzanne Shusterman, Karen Wright, Andrew E. Place, Puja J. Umaretiya, Ketki Bhushan, Steven G. DuBois, Kevin Campbell

**Affiliations:** ^1^ Harvard Medical School Boston Massachusetts USA; ^2^ Dana‐Farber/Boston Children's Cancer and Blood Disorders Center and Harvard Medical School Boston Massachusetts USA; ^3^ Division of Pediatric Hematology/Oncology University of Texas Southwestern Medical Center Dallas Texas USA; ^4^ Children's Mercy Hospitals and Clinics Kansas City Missouri USA

**Keywords:** clinical trial, oncology, pediatric, phase I, response, toxicity

## Abstract

**Background:**

Phase 1 or phase 1/2 trials are a first step in pediatric cancer drug development. Currently, there is a paucity of information regarding contemporary outcomes for pediatric patients enrolled in these trials. We describe characteristics and outcomes of patients enrolled in pediatric phase 1 clinical trials over a 9‐year period at a single institution.

**Methods:**

We queried our clinical trials management system to generate a list of patients enrolled and treated on pediatric phase 1 or phase 1/2 trials from 2011 to 2019. We collected baseline demographics, clinical data, efficacy, and safety endpoints post‐enrollment including: time to death, objective response to therapy, duration on therapy, need for dose modification, and occurrence of dose‐limiting toxicity. Overall survival was calculated using Kaplan–Meier methods.

**Results:**

A total of 224 unique patients accounted for 259 enrollments and 242 treatment episodes. The median age at enrollment was 11 years (range 0–27 years) and 56.2% were male. The majority were White (85.7%) and Non‐Hispanic (88.2%). English was the primary language for 86.3% of patients, and 54.9% had private insurance. Solid tumors were the most common malignancy (41.0%), followed by brain tumors (34.1%), and hematologic malignancies (24.9%). Among treatment episodes, 49.3% received targeted monotherapy. After first enrollment, 27.6% of patients had an objective response to therapy (52.9% for hematologic malignancies, 20.5% for brain tumors, and 15.8% for solid tumors). The median duration of therapy was 1.5 months. Median overall survival from first enrollment for 218 patients treated with available vital status was 13.1 months. Toxicity outcomes included 27 patients (11.2%) requiring dose modification and 22 patients (9.0%) having a DLT.

**Conclusions:**

Overall survival is poor for patients in pediatric oncology early phase trials, despite approximately a quarter having an initial response. These data are informative for discussions between providers and families regarding outcomes after phase 1 trial participation.

## 1 Introduction

Currently, the 5‐year overall survival rate for childhood cancer is close to 80% in high income countries; thus, nearly 20% of children succumb to their disease, or complications of treatment, within a short time [1]. Death from recurrence or death from a subsequent malignancy is the two most common causes of mortality in patients who survive 5 years from initial diagnosis. Additionally, 80% of patients who survive at least 5 years from initial diagnosis have at least one chronic medical condition [2]. In this context, novel therapies are needed to improve both short‐term and long‐term outcomes for children with cancer.

Clinical trials serve as the backbone for the advancement of new drugs or novel combinations. The first step in the clinical trial process has traditionally been phase 1 or combined phase 1/2 trials [3]. These trials most commonly enroll patients with relapsed/refractory or very rare cancers and have a primary objective of demonstrating the safety of the dosing strategy to advance to future trials [4]. For many patients who participate in early‐phase trials, there may be a lack of alternative therapeutic options, underscoring the critical role these trials play in offering potential new avenues of treatment. However, while phase 1 trials offer access to novel therapies for patients with difficult‐to‐treat cancers, outcomes are generally poor, and the act of enrolling in a phase 1 trial is in itself a major decision for patients and families [5].

Prior investigations of outcomes in early phase pediatric trials, largely before the era of genomically informed trials, have been reported. One study of 69 pediatric phase 1 trials enrolling 1973 patients between 1990 and 2004 found an overall objective response rate (ORR) of 9.6% and a dose‐limiting toxicity (DLT) rate of 24% [6]. Another evaluation of 248 phase 1 pediatric participants found a similar ORR of 9.3% [7]. Lastly, a single institution study including 106 patients in phase 1 trials reported an ORR of 12% and a DLT rate of 12.3% [8]. These reports have not addressed the extent to which early phase trial participants are representative of the broader sociodemographic features of children with cancer, though prior reports note disparities in phase 1 cancer trial enrollment in medical oncology [9]. In this context, the goals of our study were to determine the demographic features of cancer patients enrolling in pediatric phase 1 clinical trials and to provide more contemporary clinical outcomes by analyzing a 9‐year period of enrollment at a large single institution.

## 2 Methods and Materials

### 2.1 Patient Population

We conducted a retrospective cohort study of patients who were enrolled in a pediatric oncology cancer‐directed phase 1 or the phase 1 portion of a phase 1/2 trial at Dana‐Farber Cancer Institute (DFCI) or Boston Children's Hospital (BCH) with an enrollment date between January 1st, 2011 and December 31st, 2019. While DFCI and BCH are separate institutions, they collaborate in caring for pediatric oncology patients, and thus our study represents a single center cohort. These dates were chosen to provide the most up‐to‐date look prior to the impact of the COVID‐19 pandemic on clinical trial participation. The cohort includes all enrolled patients aged 0–30 years at enrollment (age range of our clinical practice) during the study period regardless of sex or underlying cancer diagnosis. Only accruals through pediatric oncology were included, and accruals of patients aged 18–30 years to medical oncology trials were excluded. Patients treated on trials containing only supportive care medications or trials for non‐oncologic indications were excluded.

### 2.2 Case Identification and Data Extraction

We performed a systematic search of DFCI's OnCore clinical trials system to generate a list of all pediatric patients who were enrolled on phase 1 or the phase 1 portion of phase 1/2 trials during our study period. We next performed a review of the electronic medical record to verify whether each patient was enrolled and then treated on a phase 1 or phase 1/2 clinical trial. We collected the following demographic and clinical data for each patient from the electronic medical record: race, ethnicity, primary language, sex, age at enrollment, type of insurance, disease status, diagnosis at time of enrollment, time from initial diagnosis to first phase 1 accrual, prior trial participation, stage at diagnosis and enrollment, enrollment based upon genomic or histologic criteria, relapsed vs. refractory disease, survival post‐enrollment, type of therapy, objective response to trial therapy, duration on therapy, participation in multiple phase 1 trials, need for dose modification throughout therapy, and occurrence of DLT throughout therapy. Type of insurance at the time of trial enrollment was classified as public [defined as sole coverage by Medicaid or Children's Health Insurance Program (CHIP)], private, international, or unknown for analysis. Type of therapy was classified as cytotoxic, cytotoxic + targeted, targeted monotherapy, targeted combination, and other (which included: stem cell therapy, immunotherapy, radiotherapy, and other biologic agents that did not clearly fall into the defined categories). For patients who enrolled on a trial but were not subsequently treated on trial, we collected only demographic information.

In order to generate a comparator group of pediatric oncology patients to compare demographic features, we queried DFCI's OnCore system to obtain demographic data from patients aged 0–30 years who enrolled on pediatric oncology phase 2, phase 2/3, phase 3, and feasibility/pilot clinical trials during our study period. It is possible that the same patient may have been included in both cohorts if a patient enrolled on both a phase 1 or phase 1/2 trial and a non phase 1 or phase 1/2 trial during the investigated time period.

### 2.3 Statistical Considerations

We analyzed demographic data for enrolled patients using descriptive statistics, with each patient represented only once even if they ultimately enrolled in multiple phase 1 or phase 1/2 clinical trials. We analyzed response, duration of therapy, and toxicity data for treated patients in the cohort using descriptive statistics, with each treatment episode represented once (i.e., a patient who was treated on multiple trials contributed outcome data from each trial). Finally, we used Kaplan–Meier methods to describe overall survival from the time of first treatment on a phase 1 or phase 1/2 clinical trial, with each patient represented only once even if they ultimately enrolled in multiple phase 1 or phase 1/2 clinical trials.

## 3 Results

### 3.1 Patient Characteristics

**FIGURE 1:**
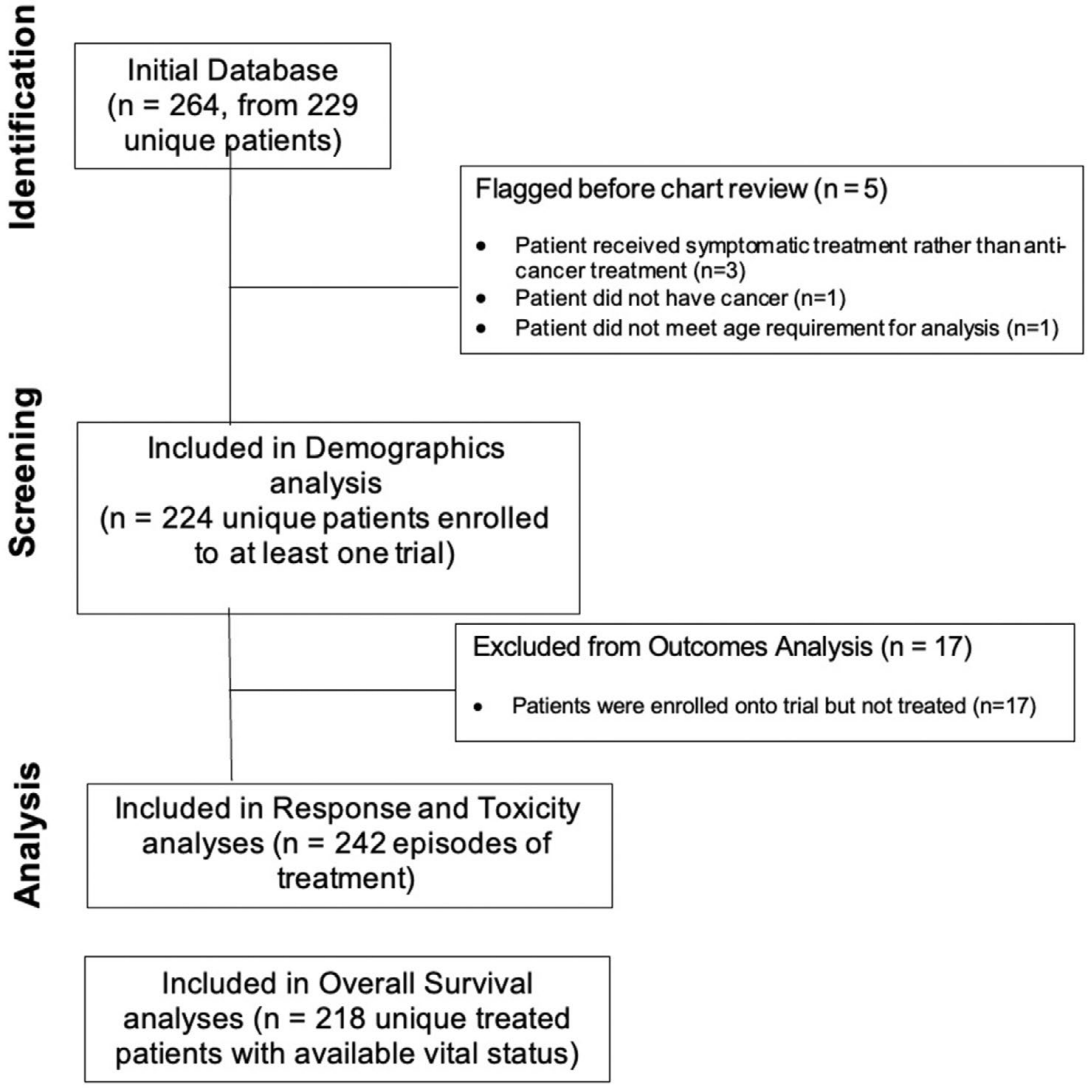
CONSORT diagram.

Of 264 identified enrollments to phase 1 or phase 1/2 clinical trials, five were not included due to: 3 patients receiving treatment on supportive care trials, 1 patient not having a cancer diagnosis, and 1 patient not meeting the age requirement for inclusion in the cohort. The 259 enrollments remaining represent 224 unique patients. Of these 224 patients, 193 enrolled on one trial and 31 patients enrolled on more than one trial (Figure 1).

The baseline characteristics of 224 patients who enrolled in at least one trial are summarized in Table 1. A majority of patients (126, 56.2%) were male, and the median age at trial enrollment was 11 years (range 0–27), with 78.6% of patients having an age at enrollment between 2 and 18 years. The majority of patients were White (69.6%) and Non‐Hispanic (76.8%). The majority of patients spoke English as their primary language (81.7%). Private insurance was most common (54.9%), followed by public insurance (24.6%) and international insurance (4.0%). Forty percent of patients had solid tumors, followed by CNS tumors (33.0%) and hematologic malignancies (24.1%). Table S1 further delineates the diagnosis of patients with brain tumors. The majority of patients enrolled in trials of single agent targeted therapies (49.3%), followed by cytotoxic only (17.5%), other (15.3%), cytotoxic and targeted (14.4%), and targeted combination therapy (3.6%). The distribution of therapy types by disease types is shown in Table S2. Most patients were enrolled in phase 1 trials based on histologic subtype (86.0%) rather than genomic features (13.1%). The median time from initial diagnosis to enrollment was 23.7 months (range 0.2–188.7).

**TABLE 1:** Characteristics of 224 unique patients *enrolled* in phase I trials.

Patient characteristic	*N* (%)
Sex	
Female	98 (43.8)
Male	126 (56.2)
Age at enrollment	
< 2 years	13 (5.8)
2–< 18 years	176 (78.6)
18+ years	35 (15.6)
Median	11 (range 0–27)
Race	
Asian	15 (6.7)
Black	11 (4.9)
White	156 (69.6)
Unknown	42 (18.8)
Ethnicity	
Hispanic	23 (10.3)
Non‐Hispanic	172 (76.8)
Unknown	29 (12.9)
Primary language	
English	183 (81.7)
Other	29 (12.9)
Unknown	12 (5.4)
Type of insurance	
Public	55 (24.6)
Private	123 (54.9)
International	9 (4.0)
Unknown	37 (16.5)
Disease status	
Refractory	98 (43.8)
Relapse	124 (55.4)
Unknown	2 (0.9)
Diagnosis	
Hematologic malignancy	54 (24.1)
Brain tumor	74 (33.0)
Solid tumor (non‐brain)	89 (39.7)
Other, NOS	7 (3.1)
Stage at diagnosis^a^	
Localized	68 (39.3)
Metastatic	102 (59.0)
Unknown	3 (1.7)
Therapy type of first trial enrolled	
Cytotoxic only	39 (17.5)
Cytotoxic + targeted	32 (14.4)
Targeted monotherapy	110 (49.3)
Targeted combination therapy	8 (3.6)
Other	34 (15.3)
Enrollment criteria of first trial	
Genomic	29 (13.1)
Histologic	191 (86.0)
Unknown	2 (0.9)
Months from diagnosis to first trial enrollment	
Median (Range)	23.7 (0.2–188.7)

^a^ Does not include patients with leukemia.

Demographic features for a comparative group of 1270 pediatric patients enrolled to any phase 2 trial, phase 2/3 trial, phase 3 trial, or feasibility/pilot study at our center during the same study period are shown in Table S3. The majority of these patients (671, 53.0%) were male. Age of enrollment between 2 and 18 years was most common (77.0%), followed by 18 years and older (14.2%) and < 2 years (8.8%). The majority of patients were white (66.3%) and Non‐Hispanic (72.8%). Primary language and insurance data were not available.

### 3.2 Efficacy and Toxicity Outcomes

Of the 259 unique patient enrollments, 242 received protocol therapy and are the focus of analyses of outcomes of therapy (Table 2). The median duration of therapy for all patients was 1.5 months. An objective response was observed in 61/242 (25.2%) episodes of treatment on trials. The response rate following initial phase 1 trial participation was 27.6%, compared to 9.4% following subsequent trial participation (Table 3). Among first episodes of trial treatment, the response rate was 54.2% for hematologic malignancies, 21.1% for brain tumors, and 18.8% for solid tumors. Response rates by therapy type following initial phase 1 participation were 54.4% for patients receiving cytotoxic only therapy, 23.3% for patients receiving cytotoxic plus targeted therapy, 22.0% for patients receiving targeted monotherapy, and 14.3% for patients receiving targeted combination therapy (Table 4). Among patients for whom enrollment based upon genomic or histologic criteria was known (*n* = 220), the ORR was 34.5% for those enrolled based on genomic criteria and 24.6% for those enrolled based on histologic criteria (*p* = 0.258).

**TABLE 2:** Outcome variables for 242 enrollments from 224 patients treated on phase 1 trials.

Reporting characteristic	*n* (%)
Objective response to trial therapy	
No	181 (74.8)
Yes	61 (25.2)
Need for dose modification while on trial	
No	215 (88.8)
Yes	27 (11.2)
Occurrence of a dose‐limiting toxicity	
No	220 (91.0)
Yes	22 (9.0)

**TABLE 3:** Outcomes stratified by initial vs. subsequent trial and diagnosis for 242 patients *treated* on phase 1 trials.

	All	Hematologic malignancy	Solid tumor	Brain tumor	Unknown
Objective response					
Initial trial					
No	152 (72.4)	22 (45.8)	69 (81.2)	56 (78.9)	5 (83.3)
Yes	58 (27.6)	26 (54.2)	16 (18.8)	15 (21.1)	1 (16.7)
Subsequent trial					
No	29 (90.6)	2 (66.7)	16 (100)	10 (83.3)	1 (100)
Yes	3 (9.4)	1 (33.3)	0 (0)	2 (16.7)	0 (0)
Dose limiting toxicity					
Initial trial					
No	191 (90.1)	45 (91.8)	73 (83.9)	68 (97.1)	5 (83.3)
Yes	21 (9.9)	4 (8.2)	14 (16.1)	2 (2.9)	1 (16.7)
Subsequent trial					
No	31 (96.9)	3 (100)	15 (93.7)	12 (100)	1 (100)
Yes	1 (3.1)	0 (0)	1 (6.3)	0 (0)	0 (0)
Dose modification					
Initial trial					
No	189 (88.3)	48 (96.0)	70 (80.5)	65 (91.5)	6 (100)
Yes	25 (11.7)	2 (4.0)	17 (19.5)	6 (8.5)	0 (0)
Subsequent trial					
No	30 (93.8)	3 (100)	15 (93.7)	11 (91.7)	1 (100)
Yes	2 (6.3)	0 (0)	1 (6.3)	1 (8.3)	0 (0)

**TABLE 4:** Outcomes stratified by initial vs. subsequent trial and therapy type for patients *treated* on phase 1 trials.

	All	Cytotoxic only	Cytotoxic + Targeted	Targeted monotherapy	Targeted combination	Other
Objective response						
Initial trial						
No	152 (72.4)	18 (48.6)	23 (76.7)	85 (78.0)	6 (85.7)	20 (74.1)
Yes	58 (27.6)	19 (54.4)	7 (23.3)	24 (22.0)	1 (14.3)	7 (25.9)
Subsequent trial						
No	29 (90.6)	3 (100)	7 (100)	18 (94.7)	0 (0)	1 (50)
Yes	3 (9.4)	0 (0)	0 (0)	1 (5.3)	1 (100)	1 (50)
Dose limiting toxicity						
Initial trial						
No	191 (90.1)	36 (97.3)	26 (86.7)	95 (88.0)	7 (87.5)	27 (93.1)
Yes	21 (9.9)	1 (2.7)	4 (1.3)	13 (12.0)	1 (12.5)	2 (6.9)
Subsequent trial						
No	31 (96.9)	3 (100)	7 (100)	18 (94.7)	1 (100)	2 (100)
Yes	1 (3.1)	0 (0)	0 (0)	1 (5.3)	0 (0)	0 (0)
Dose modification						
Initial trial						
No	189 (88.3)	35 (94.6)	24 (80.0)	95 (87.2)	7 (87.5)	28 (93.3)
Yes	25 (11.7)	2 (5.4)	6 (20.0)	14 (12.8)	1 (12.5)	2 (6.7)
Subsequent trial						
No	30 (93.8)	3 (100)	6 (85.7)	18 (94.7)	1 (100)	2 (100)
Yes	2 (6.2)	0 (0)	1 (14.3)	1 (5.3)	0 (0)	0 (0)

Dose‐limiting toxicity was observed in 22 (9%) patients, with 14 of those patients requiring dose modifications. An additional 13 patients without DLT also required dose modifications while on trial, for a total of 27 (11.2%). Table 3 shows the rates of DLT and dose modifications were 9.9% and 11.7%, respectively, after initial trial participation and 3.1% and 6.3% after subsequent trial participation. Rates of DLT and dose modifications after initial trial participation were both highest in patients with solid tumors (16.1% and 19.5%) compared to hematologic malignancies (8.2%, 4.0%) and brain tumors (2.9%, 8.5%). Rates of DLT and dose modifications in subsequent trials were also highest in patients with solid tumors (6.3% and 6.3%) compared to hematologic malignancies (0%, 0%) and brain tumors (0%, 8.3%). When stratified by therapy type, higher rates of DLT and dose modifications were seen in patients treated with targeted therapies compared with cytotoxic therapy only (Table 4).

**FIGURE 2:**
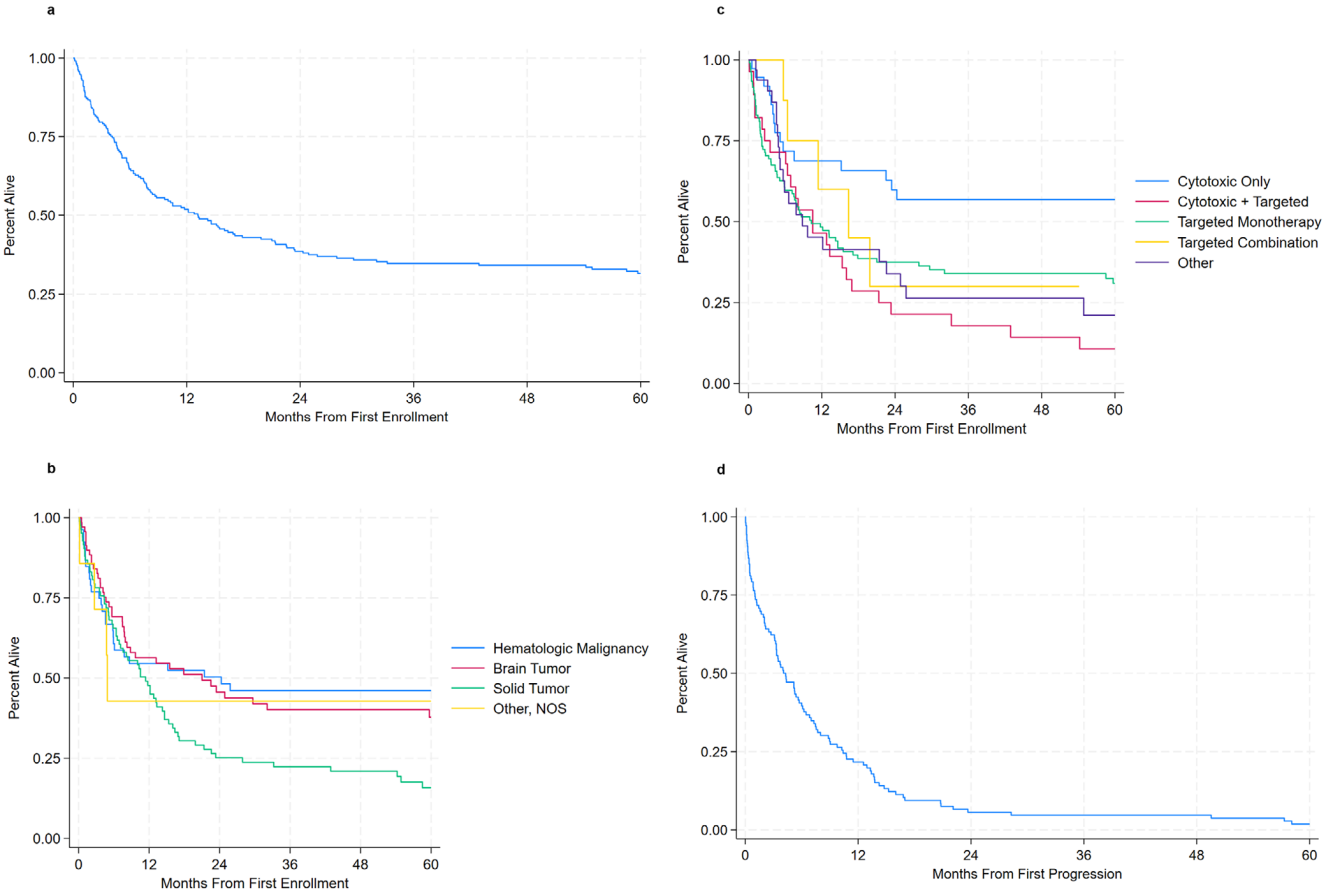
(a) Overall survival from first enrollment (*n* = 218). (b) Overall survival from first enrollment by diagnosis (*n* = 218). (c) Overall survival from first enrollment by therapy type (*n* = 218). (d) Time from disease progression to death (*n* = 135).

Median overall survival from first enrollment for 218 patients treated with available vital status was 13.1 months (Figure 2a). Notably, more than 25% of patients were alive 3+ years from the time of first enrollment. Patients with solid tumors had particularly poor outcomes, with a median overall survival from first enrollment of 10.5 months (Figure 2b), followed by 17.9 months for patients with brain tumors and 24.3 months for patients with hematologic malignancies. Assessed by therapy type received at first enrollment, patients treated with targeted monotherapy had the shortest median overall survival from first enrollment at 8.4 months, followed by those receiving cytotoxic plus targeted therapy at 10.5 months and targeted combination therapy at 19.8 months (Figure 2c). For the 135 patients who progressed on trial, the median time from progression to death was 8.1 months (Figure 2d).

## 4 Discussion

In this study, we present a comprehensive analysis of pediatric cancer patients who were enrolled in a phase 1 or phase 1/2 trial and received therapy at our center over a 9‐year period. We found differential responses according to disease type, therapy type, and initial versus subsequent treatment in phase 1 trials. We report contemporary data regarding the duration of therapy and of overall survival once a patient has enrolled in a phase 1 trial. We also note a low probability of developing significant toxicity while on these trials, with low rates of DLT or dose modification. We observed this cohort to have broadly similar demographic features when compared to the population of pediatric oncology patients enrolled in later phase clinical trials at our center. Taken together, our data allow for improved understanding for both providers and patients/parents of expectations of outcomes once enrolled in phase 1 studies.

Previous studies on early phase pediatric trials reported ORR of 9%–12% and DLT rates of 12%–24% [6, 7, 8]. Encouragingly, our study from a more contemporary cohort reported an ORR of 25% and DLT rate of 9%. These findings may reflect more precise patient to therapy matching, the use of innovative treatments, and advancements in supportive care. Further, prior studies have highlighted differences in pediatric oncologic outcomes based on initial diagnosis [10]. From this study, looking specifically at outcomes after treatment on phase 1 or phase 1/2 studies, we found that pediatric patients with hematologic malignancies had a higher ORR and overall survival during their initial trial compared to patients with CNS and solid tumors. Notably, a similar pattern was seen with objective response to subsequent phase 1 or phase 1/2 trial, with higher likelihood of response to therapy seen in patients with hematologic malignancies, followed by CNS and then solid tumors. Interpreting these differences is challenging as patients with hematologic malignancies may have been more likely to receive cytotoxic agents while those with CNS or solid tumors may have more often received targeted therapies. Additional work is needed to determine if treatment type explains the observed differences in response and toxicity. Although cure rates for childhood solid tumors have increased by as much as 50% since the 1970s [11], our findings underscore the persistent difficulty in controlling these diseases after relapse and the urgent need for more effective salvage options.

We found that ORRs are much lower for patients on subsequent phase 1 trials compared to patients on their initial trial, potentially reflecting acquired therapy resistance or the inability of patients with the most aggressive disease to enroll in more than one trial. Across all diagnoses, cytotoxic therapy was associated with the highest response rates, and responses were most favorable in initial trial participation and in hematologic malignancies. These trends highlight the importance of therapy selection and disease context in the design and interpretation of early phase pediatric trials.

With the advent of precision oncology, the landscape of treating pediatric cancers is shifting, with movement away from histologic selection and cytotoxic agents and towards genomic criteria and targeted agents. Despite a push to enroll patients based on genomic criteria, our study found that only 13.2% of patients enrolled based upon genomic criteria. This finding may reflect a paucity of available trials with therapies aligned with the genomic features seen in pediatric cancers. In our cohort, patients enrolled based on genomic criteria demonstrated a numerically higher ORR compared with those enrolled based on histologic criteria. Previous data demonstrated that, in phase 1 pediatric solid tumor trials, ORRs were significantly higher for patients treated with cytotoxic versus targeted agents [12]. Further, strong biological rationale and clinical experience suggest multiple agents will be more efficacious than monotherapy for most diseases and may overcome resistance mechanisms and increase synergy [13]. Our results demonstrated that patients on trials with cytotoxic‐only treatments had the highest objective response and overall survival rates. The explanation for this observation is not clear, but highlights the ongoing important role of chemotherapy in managing many pediatric cancers.

The majority of patients enrolled on phase I trials were White (69.6%) and Non‐Hispanic (76.8%), comparable to the racial and ethnic characteristics of patients enrolled on phase 2, phase 2/3, phase 3, or feasibility/pilot combined trials, who identified as White (66.3%) and Non‐Hispanic (72.8%). A prior analysis of phase 1 trials across the Children's Oncology Group and Pediatric Brain Tumor Consortium previously noted underrepresentation of Hispanic children relative to other groups [14]. Despite evidence that Black and Hispanic children are more likely to relapse or die from cancer [15, 16], their representation in clinical trials remains disproportionately low. Only a small percentage of patients in both cohorts were Black (~5%) or Hispanic (~10%). Furthermore, in our cohort, there was a significant number of patients whose demographic information was unknown, which complicates our understanding of true representation. Given the heightened risks these populations face, they should be overrepresented in phase 1 trials. Finally, our comparator group only includes patients at our center who participated in later phase trials. Therefore, this population may not be representative of the demographics of our entire clinical population that also includes patients and families who were not offered trial participation, declined participation, or did not meet eligibility criteria. Ensuring representative participation across sociodemographic groups is essential, not only to enhance the generalizability of trial findings but also to ensure that the risks and benefits of early phase trials are equitably shared.

While we present an original study of patient characteristics and outcomes for pediatric phase 1 oncology trials that provides current insights, several limitations must be highlighted when considering this study. Our study represents data from a large academic medical center, which allowed for a detailed analysis of patient demographics and clinical characteristics. However, this patient population may not be generalizable to other centers. Further, due to the retrospective nature of chart review, we note incomplete or missing data in our analysis, and responses were not centrally reviewed. We did not collect data on the number of lines of prior therapy, and future studies should assess the impact of the extent of prior treatment on toxicity and efficacy endpoints. Survival comparisons should, therefore, be interpreted with caution given the heterogeneity in underlying diseases, treatment histories, and extent of prior therapy, which were not systematically captured in this analysis. Moreover, some demographic features of interest were not available for the comparator group, such as primary language and insurance type, motivating future studies in this area to ensure the representativeness of pediatric oncology clinical trial participants. Finally, we acknowledge the limitation of not having data for patients who qualified for but did not participate in early phase trials, which restricts our ability to understand the extent to which outcomes might differ between children with advanced cancer who participated in trials versus received best available therapy off trial.

Overall, our study demonstrates that clinical outcomes on pediatric cancer phase 1 trials vary by initial diagnosis, with patients with hematologic malignancies demonstrating the highest objective response and overall survival rates after phase 1 trial participation. While trial therapy was generally tolerable, the majority of patients did not have an objective response. These findings provide immediate insight to both providers and patients/families about disease course, overall response to therapy, and outcomes following progression, which will allow for more informed decision‐making when discussing clinical trial enrollment. Our results are also more broadly motivating for the field to develop more effective therapies for patients with relapsed/refractory disease.

## Author Contributions

**Avina Rami:** investigation, writing – original draft, writing – review and editing, validation, visualization, formal analysis, data curation, funding acquisition. **Kira Bona:** writing – review and editing. **Suzanne Shusterman:** writing – review and editing. **Karen Wright:** writing – review and editing. **Andrew E. Place:** writing – review and editing. **Puja J. Umaretiya:** writing – original draft, writing – review and editing. **Ketki Bhushan:** writing – review and editing. **Steven G. DuBois:** writing – review and editing, validation, visualization, formal analysis, data curation, funding acquisition, conceptualization, methodology, project administration, supervision. **Kevin Campbell:** writing – original draft, writing – review and editing, validation, visualization, formal analysis, data curation, conceptualization, methodology, project administration.

## Ethics Statement

This study was approved by the Institutional Review Board of the Dana‐Farber Cancer Institute under reference number 22–032. Given that this was a retrospective chart review involving no more than minimal risk to participants, the requirement for informed consent was waived by the IRB.

## Conflicts of Interest

S.G.D. reports travel expenses from Loxo Oncology, Roche, and Salarius and consulting fees from Amgen, Bayer, Inhibrx, and Jazz Pharmaceuticals.

## Supporting information


**Tables S1–S3:** cam471222‐sup‐0001‐TableS1‐S3.docx.

## Data Availability

The data that support the findings of this study are available on request from the corresponding author. The data are not publicly available due to privacy or ethical restrictions.
