# Supplementary material for: Contemporary Characteristics and Outcomes of Pediatric Oncology Patients Participating in Early Phase Clinical Trials
Source: Cancer Med. 2025 Sep 8;14(17):e71222. doi: 10.1002/cam4.71222 (PMC12415703; doi:10.1002/cam4.71222)
Supplement: Supplementary file 1 — Tables S1–S3: cam471222‐sup‐0001‐TableS1‐S3.docx. [file CAM4-14-e71222-s001.docx]

**Supplemental Table 1.** Specified diagnosis of 87 patients with brain tumors ***enrolled*** in Phase I or Phase I/II Trials

| **Diagnosis** | **N (%)** |
| --- | --- |
| High-grade glioma  Low-grade glioma  ATRT  Medulloblastoma  NF1 lesion  Ependymoma  Astrocytoma  Pineoblastoma  Neuro-oncologic lesion NOS | 33 (37.9)  22 (25.8)  9 (10.3)  9 (10.3)  5 (5.7)  4 (4.6)  2 (2.3)  2 (2.3)  1 (1.1) |

**Supplemental Table 2.** Therapy modalities administered to 224 unique patients by cancer diagnosis.

|  | **Cytotoxic Only** | **Cytotoxic + Targeted** | **Targeted Monotherapy** | **Targeted Combination** | **Other** |
| --- | --- | --- | --- | --- | --- |
| **Hematologic Malignancies** | 25 (46.3) | 3 (5.6) | 15 (27.8) | 0 | 11 (20.4) |
| **Brain Tumors** | 12 (16.2) | 3 (4.1) | 49 (66.2) | 1 (1.4) | 9 (12.2) |
| **Solid Tumors** | 2 (2.5) | 26 (29.2) | 42 (47.2) | 7 (7.9) | 12 (13.5) |
| **Other (NOS)** | 0 | 0 | 4 (57.1) | 0 | 3 (42.9) |

**Supplemental Table 3.** Characteristics of 1270 patients ***enrolled*** in Phase II, Phase II/III, Phase III or Feasibility/Pilot Combined Trials

| **Patient Characteristic** | **N (%)** |
| --- | --- |
| **Sex**  Female  Male  Unknown | 595 (47.0)  671 (53.0)  4 |
| **Age at Enrollment**  <2 years  2 - <18 years  18+ years | 112 (8.8)  924 (77.0)  207 (14.2) |
| **Race**  Asian  Black  White  Other  Unknown | 42 (3.3)  69 (5.4)  842 (66.3)  110 (8.7)  207 (16.3) |
| **Ethnicity**  Hispanic  Non-Hispanic  Unknown | 139 (10.9)  924 (72.8)  207 (16.3) |
